# Impact of Genetic Variations and Epigenetic Mechanisms on the Risk of Obesity

**DOI:** 10.3390/ijms21239035

**Published:** 2020-11-27

**Authors:** Martina Chiurazzi, Mauro Cozzolino, Roberta Clara Orsini, Martina Di Maro, Matteo Nicola Dario Di Minno, Antonio Colantuoni

**Affiliations:** 1 Department of Clinical Medicine and Surgery, University of Naples “Federico II”, 80131 Naples, Italy; roberta.orsini@alice.it (R.C.O.); martinadimaro@hotmail.it (M.D.M.); colantuo@unina.it (A.C.); 2 Department of Obstetrics, Gynecology and Reproductive Sciences, Yale School of Medicine, New Haven, CT 06511, USA; mauro.cozzolino@yale.edu; 3 Rey Juan Carlos University, Calle Tulipán, 28933 Móstoles, Spain; 4 IVIRMA, IVI Foundation, Avenida Fernando Abril Martorell, 106, 46026 Valencia, Spain; 5 Department of Translational Medical Sciences, University of Naples “Federico II”, 80131 Naples, Italy; mnd.diminno@gmail.com

**Keywords:** obesity, monogenic obesity, syndromic obesity, polygenic obesity, epigenetics

## Abstract

Rare genetic obesity disorders are characterized by mutations of genes strongly involved in the central or peripheral regulation of energy balance. These mutations are effective in causing the early onset of severe obesity and insatiable hunger (hyperphagia), suggesting that the genetic component can contribute to 40–70% of obesity. However, genes’ roles in the processes leading to obesity are still unclear. This review is aimed to summarize the current knowledge of the genetic causes of obesity, especially monogenic obesity, describing the role of epigenetic mechanisms in obesity and metabolic diseases. A comprehensive understanding of the underlying genetic and epigenetic mechanisms, with the metabolic processes they control, will permit adequate management and prevention of obesity.

## 1. Introduction

Obesity, a complex and multifactorial disease associated with excessive adiposity or body fat, currently affects over a third of the world’s population [1,2]. Obesity is closely related to a significant increase in the morbidity risk of chronic diseases, such as disability, depression, type 2 diabetes, hypertension, cardiovascular diseases, cancers, and mortality, thus representing a serious public health problem [3]. According to the World Health Organization (WHO), the body mass index (BMI) is used as a tool to assess overweight or obesity; a BMI ≥ 40 kg/m^2^ is characteristic of severe obesity [4,5]. Obesity is a consequence of an energy imbalance between caloric intake and energy expenditure, leading to a positive energy balance with a consequent increase in body weight [6,7]. Factors both hereditary or genetic, family history, socioeconomic and sociocultural conditions are considered risk factors for obesity [1]. Much evidence, through the study of genes strongly involved in the central or peripheral regulation of energy balance, including variants in leptin (LEP), leptin receptor (LEPR), proopiomelanocortin (POMC), neuropeptide Y (NPY), melanocortin receptor (MC4R), and the gene associated with fat mass and obesity (FTO), suggests a genetic component, contributing to 40–70% of obesity. However, the roles of genes in the processes leading to obesity are still unclear [8,9]. Furthermore, the genes that could influence an individual predisposition to gain weight are still unknown. Modern gene technology has improved our understanding of the molecular mechanisms involved in body weight regulation by identifying poorly known genetic aberrations [10,11]. Obesity induced by genetic factors can be classified into monogenic, caused by a single genetic mutation, syndromic, associated with other phenotypes, such as abnormalities of neurological development or organs/systems malformations, and finally, polygenic caused by the mutation of a large number of genes [10,12] (Table 1). In addition, in the last years, a link between epigenetic modifications and metabolic health in humans has been found; research has been focused on the role of epigenetics and on metabolic disorders associated with obesity [13,14]. This review is aimed to summarize the current knowledge of the genetic causes of obesity, especially monogenic obesity, with the purpose to describe the role of epigenetic mechanisms in obesity and metabolic diseases.

## 2. The Role of Hormones in Appetite and Weight Regulation

The central nervous system (CNS), in particular the hypothalamus, plays a fundamental role in regulating appetite and energetic homeostasis in response to changes in peripheral circulating signals, such as hormones and nutrients. The reception and integration of these signals occur mainly in the arcuate nucleus (ARC), which contains two distinct populations of neurons, one releasing agouti-related protein (AgRP) and the other releasing proopiomelanocortin (POMC). These neurons are involved in the regulation of energy homeostasis, sensing and integrating numerous metabolic signals [15]. It has been observed that in the hypothalamus, leptin binds its receptor (LepR-b) and triggers a signaling pathway, which induces STAT3 stimulation with subsequent activation of POMC neurons and NPY/AgRP/GABA neurons inhibition, exerting an anorexigenic effect. Leptin is one of the main components of the physiological system involved in the regulation of body weight [16]. Furthermore, several data have shown that insulin binding to the insulin receptor substrate (IRS) exerts the same anorexigenic effect of leptin by activating a phosphorylation cascade through phosphatidylinositide-3-kinase (PI3K) in POMC neurons [17]. Experimental and human studies have shown that cholecystokinin (CCK) reduces food intake dose-dependently in response to meal initiation. CCK is synthesized both in the gastrointestinal tract and in the hypothalamus, representing the most abundant neuropeptide in the CNS. At the hypothalamic level, indeed, CCK, binding to CCK-B receptor and interacting with NPY, induces satiety. CCK has been studied as a potential therapeutic substance for the management of obesity [15]. Tyrosine–tyrosine polypeptide (PYY) and glucagon-like peptide 1 (GLP-1) are produced by L cells in the small intestine, leading to an anorexigenic action. In response to food ingestion, PYY plasma concentration increases, signaling food ingestion from the intestine to the appetite-regulating system in the brain: PYY mediates its effects by binding to its Y2 receptor [18]. GLP-1, on the other hand, acts by binding to a membrane receptor, GLP-1-R, whose activation stimulates the production of cyclic adenosine monophosphate (cAMP) by the enzyme adenylyl cyclase. This mechanism triggers the transmission of intracellular signaling [19]. Conversely, ghrelin binds its receptor, the growth hormone receptor (GHSR), inducing the activation of AgRP neurons and the inhibition of POMC neurons, exerting orexigenic effects [15].

## 3. Monogenic Obesity

**Figure 1:**
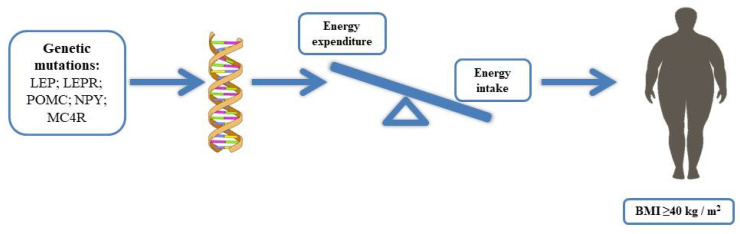
Obesity is caused by an energy imbalance between caloric intake and energy expenditure, leading to a positive energy balance with a consequent increase in body weight; some genes strongly involved in the central or peripheral regulation of energy balance, including variants of leptin (LEP), leptin receptor (LEPR), proopiomelanocortin (POMC), neuropeptide Y (NPY), melanocortin receptor (MC4R), and the gene associated with fat mass and obesity (FTO), have suggested that the genetic component can contribute to 40–70% of obesity.

Monogenic obesity is caused by a mutation or deficiency in a single gene [20]. Mutations in genes that have a physiological role in the hypothalamic leptin–melanocortin energy balance system, such as mutations in leptin, leptin receptor, POMC, prohormone 1/3 convertases (PC1/3), MC4R, are related to the currently known monogenic forms of obesity (Figure 1). However, three genes, i.e., SIM1, BDNF, and tropomyosin-related kinase B (TRKB), when mutated, cause obesity, although the exact mechanisms by which these genetic defects lead to obesity are still unknown [5,21,22]. Mutations of the indicated genes are extremely rare, and new studies are essential to investigate these genetic variants with the aim to clarify the mechanisms and promote effective strategies to control and counteract the progression of specific clinical phenotypic features. Furthermore, new techniques, such as mass spectrometry, might reveal new available markers, as in the case of LEPR mutations, useful for early recognition of obesity risk and intervention.

### 3.1. Leptin and Leptin Receptor

Leptin is a type I cytokine encoded by the *LEP* gene and secreted mainly by adipocytes. Leptin is synthesized as an immature 167-amino acid protein, converted into a mature form after the cleavage of the 21-amino acid N-terminal peptide [10,23]. The function of leptin is to signal satiety in the hypothalamus [15,24,25]. Monogenic obesity due to homozygous mutations in the leptin gene is a rare autosomal recessive disorder, leading to severe early-onset obesity and congenital circulating leptin deficiency [21]. This type of deficiency was first identified in 1997, in two extremely obese Pakistanis. A homozygous single-base deletion at codon 133 caused a frameshift mutation, leading to a truncated leptin molecule and diminished serum leptin levels [26]. Since then, few other cases have been reported in the world with mutations in the leptin gene. All patients with this congenital leptin deficiency showed similar phenotypic manifestations, such as rapid weight gain in the first three months of life, resulting in early-onset obesity, hyperphagia, and significantly low serum leptin levels. In addition to the significant weight gain, the patients showed serious and potentially lethal bacterial infections, due to defective T cell immunity and hypogonadotropic hypogonadism, and severe obesity complications, such as hyperinsulinemia, severe liver steatosis, and dyslipidemia [21,27]. In 2019, Yupanqui-Lozno et al. reported a new leptin homozygous missense mutation in exon 3 in two severely obese Colombian sisters. The data collected indicated LEP mutation, absence of detectable circulating leptin, and severe obesity, providing evidence of the first monogenic leptin deficiency reported in North and South America [28]. Currently, the daily subcutaneous administration of recombinant human leptin is the most effective treatment used to control clinical manifestations in the case of leptin deficiency [29]. LEPR mutations, with consequent congenital leptin deficiency, also cause severe early-onset obesity, despite a normal birth weight [30]. The first identification of leptin receptor deficiency occurred in three sisters with a homozygous mutation [31]. The clinical phenotypic features included severe obesity, hypogonadotropic hypogonadism, and impaired immune function [32]. Individuals with homozygous LEPR mutations cannot be treated with recombinant leptin administration and do not exhibit low serum leptin levels [12]. In 2007, Farooqui et al. sequenced LEPR in 300 subjects with early-onset hyperphagia and severe obesity, and 8 subjects showed LEPR nonsense or missense mutations, determining receptor signaling abnormalities. These data revealed that serum leptin levels in the mutated subjects were within the range expected according to their high-fat mass, suggesting serum leptin cannot be used as a marker for leptin receptor deficiency. Moreover, patients with nonsense or missense mutations showed less severe clinical features than those with congenital leptin deficiency [32]. LEP deficiency and LEPR mutations are extremely rare; however, serum leptin level can help to distinguish between the two conditions [12].

### 3.2. Proopiomelanocortin (POMC)

POMC plays a fundamental role in the leptin–melanocortin system and represents the precursor of some biologically active proteins produced in the anterior pituitary gland and/or in the hypothalamus and skin [33,34]. A deficiency of the POMC protein causes the absence of adrenocorticotrophic hormone (ACTH ) and α-melanocyte stimulating hormone (α-MSH), known to be POMC cleavage products [35,36]. α-MSH can activate MC4R; it is involved in regulating both appetite and pigmentation. Deficiency in POMC with consequent inactivation of MC4R by α-MSH reduction causes hyperphagia, severe obesity, and red hair [31,37]. On the other hand, the lack of substrate for the synthesis of ACTH in the anterior pituitary causes adrenal insufficiency; the early recognition of adrenal insufficiency and the use of glucocorticoids are essential for the treatment of this condition [10]. Few cases have been described worldwide: in 1998, the first two patients with complete POMC deficiency were identified. One of them presented mutations in exon 3, with consequent deficiency of ACTH and α-MSH, and the other one had a mutation in exon 2, which prevented the translation of POMC; both patients showed adrenal insufficiency, red hair pigmentation, and early-onset obesity [38]. In 2008, Creemers et al. identified and characterized new mutations in the POMC gene in patients with early-onset obesity. In this study of 500 patients with severe early-onset obesity, two new heterozygous missense mutations in a region of POMC N terminus (C28F and L37F) were identified; these mutations were able to prevent POMC processing and thus the generation of bioactive products, suggesting a new molecular mechanism of human POMC deficiency [39].

### 3.3. Prohormone Convertase 1/3 and 2 (PC1/3 and PC2)

Protein convertase (PC) is a family of serine neuroendocrine endoproteases that transform precursors of inactive hormones into biologically active secreted peptides [40]. PC 1/3 and PC2, among the seven members of this family, are needed for the synthesis of many peptides involved in energy homeostasis. ProTRH, proinsulin, proglucagon, proGHRH, POMC, proNPY, and proCART represent the substrates for PC 1/3 and PC2 [21]. Three cases of PC 1/3 mutations have been reported, with high variability in the clinical phenotype [41,42]. The PC 1/3 mutation appears to be associated with severe early-onset obesity, adrenal, gonadotropic, somatotropic and thyrotropic insufficiency, malabsorption due to dysfunction of the small intestine, abnormalities of glucose homeostasis, in particular postprandial hyperglycemia, and subsequent reactive hypoglycemia [21]. Proprotein convertase subtilisine/kexin type 1 (PCSK1), which encodes 1/3 prohormone convertase (PC 1/3), was one of the first genes linked to early-onset monogenic obesity. Many genome studies of a variety of different populations have demonstrated a link between three frequent PCSK1 polymorphisms and an increased risk of obesity [43].

### 3.4. Melanocortin Receptor (MC4R)

**Figure 2:**
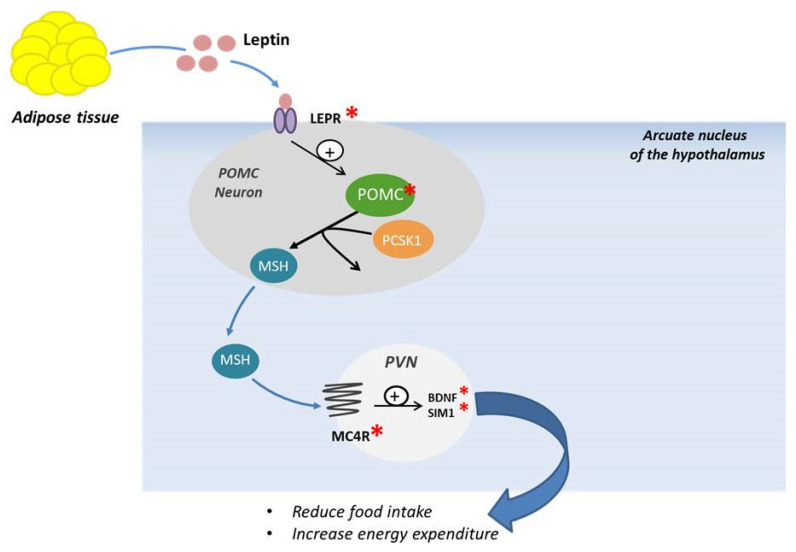
Schematic representation of POMC neuronal and melanocortin-4 receptor activation. LEPR: Leptin receptor, PCSK1: proprotein convertase subtilisine/kexin type 1, POMC: Proopiomelanocortin, MSH: melanocyte stimulating hormone, PVN: paraventricular nucleus, MC4R: Melanocortin Receptor, BDNF: Brain-derived Neurotrophic Factor, SIM1: Sim BHLH Transcription Factor1.

MC4R is a seven-transmembrane receptor coupled with G proteins, highly expressed in the hypothalamus and playing a fundamental role in the leptin–melanocortin signaling system. This receptor is largely located in the hypothalamic nuclei that control food intake through both anorexigenic and orexigenic signals; mutations of this receptor could influence the feeding behavior [15,44,45,46]. The MC4R pathway is closely linked to key proteins upstream of MC4R, such as LEP, LEPR, POMC, and PCSK1. The signaling pathway is activated by the binding of LEP to LEPR located on the surface of POMC neurons in the arcuate nucleus of the hypothalamus. Neuropeptides originated from POMC can activate MC4R to promote satiety, energy expenditure, and weight loss (Figure 2).

This activation will induce further downstream signaling with production of brain-derived neurotrophic factor (BDNF) and single-minded transcription factor 1 (SIM 1), involved in reducing food intake and modulating energy expenditure [47]. MC4R mutations, both in dominant and in recessive forms, are the most common cause of monogenic obesity known so far [21,48]. The first cases of MC4R mutation were identified in 1998 in patients affected by severe obesity and hyperphagia [49]. The obesity phenotype of MC4R heterozygous mutations carriers is highly variable [21]. Collet et al., in 2017 [50], demonstrated that setmelanotide, a potent MC4R agonist, can lead to body weight loss in obese people with MC4R deficiency, although the mechanisms of weight loss are still not clear. In addition, patients with POMC defects upstream of MC4R exhibited significantly greater weight loss with setmelanotide than patients with MC4R deficiency alone or obese controls. Further studies are required to establish an effective therapy to counteract obesity caused by this mutation [50]. A previous study carried out in patients with rare defects in the gene encoding POMC, showing obesity as well as hyperphagia, hypopigmentation, and early-onset hypocortisolism, showed that treatment with setmelanotide induced a decrease in appetite and significant weight loss [51]. In 2018, Iepsen et al. assessed the effects of GLP-1 receptor agonists (GLP-1 RA) administration, which induce weight loss and decrease appetite in patients presenting pathogenic mutations of MC4R. Their results showed that the effects of the GLP-1 RA liraglutide were independent of the MC4R pathway, suggesting that this could represent a therapeutic opportunity for patients with various forms of monogenic obesity, especially for those with MC4R mutation [52].

### 3.5. SIM1, BDNF and TRKB

Mutations in genes coding for SIM1, BDNF, and TRKB appear to be involved in the development of obesity in both mice and humans, although the regulatory mechanisms are still unknown [10,21]. SIM1 is a transcription factor located on chromosome 6 and is strongly expressed in the paraventricular nucleus (PVN) of the hypothalamus, known to play a fundamental role in regulating appetite. Heterozygous deletions or mutations in SIM1 have been associated with early-onset obesity with hyperphagia, food impulsivity, and a decrease in the total number of neurons in the PVN [21,53]. BDNF and its TRKB receptor are a family of growth factors able to regulate the proliferation, survival, and differentiation of neurons during neuronal development and of plasticity in the adult nervous system. These neurotrophins have been implicated in the regulation of food intake and body weight in animal studies. BDNF and TRKB deficiencies have been associated with early-onset obesity, even though further studies are required to clarify the role of these three genes [54].

## 4. Syndromic Obesity

Syndromic obesity includes Bardet–Biedl, Prader–Willi, Alstrom, and Smith–Magenis syndromes, characterized by obesity as the predominant phenotype and associated with disorders such as mental retardation, congenital defects in organs, dysmorphism of the limbs, or endocrine and facial dysfunction. Although numerous studies have revealed the genes or chromosomal regions involved in the etiology of many of these syndromes, their relationship with the development of obesity is still unknown [10,20,55]. Bardet–Biedel syndrome (BBS) is a rare autosomal recessive ciliopathy, characterized by retinal dystrophy, obesity, post-axial polydactyly, renal dysfunction, mental retardation, and hypogonadism. Currently, mutations in 18 different genes causing BBS have been identified [56,57]. Prader–Willi Syndrome (PWS) is the most common cause of syndromic obesity worldwide. Patients with this syndrome have severe neonatal hypotonia, eating disorders, such as anorexia and hypophagia, global cognitive impairment, behavioral abnormalities, hypotonia, delayed motor development, and hormonal deficiencies, such as growth hormone deficiency, hypothyroidism, hypogonadism, and ghrelin abnormalities. The genetic defect in PWS is the inactivation of the Prader–Willi critical region (PWCR) located in the 15q11–13 region of the paternal chromosome, while the PWCR on the maternal chromosome is epigenetically silenced through methylation, which leads to the monoallelic expression of paternal genes. The genes localized in the PWCR and expressed in the hypothalamus, involved in the syndrome, are *MKRN3* (makorin 3), *MAGEL2* (similar to MAGE 2), *NDN* (necdin), *NPAP1* (nuclear pore-associated protein 1), *SNURF*–*SNRPN* (SNRPN upstream reading frame–small nuclear ribosomal protein 1) [58]. Alstrom syndrome is another rare autosomal recessive syndrome caused by mutations in the *ALMS1* gene, which shows retinal degeneration, early-onset obesity, type 2 diabetes mellitus, and perceptive hearing loss, as observed in the case of BBS. In addition, this syndrome exhibits primary cilia dysfunction, suggesting that the ALMS1 protein located in the centrosome and ciliary basal body plays a role in the formation or maintenance of primary cilia [21,59]. Smith–Magenis syndrome (SMS) is a neurodevelopmental disorder associated with intellectual disability, sleep disturbance, early-onset obesity, and extensive behavioral deficits, caused by a heterozygous microdeletion containing retinoic acid-induced gene 1 (RAI1) or by a mutation within RAI1. Like PWS, it shows early weight gain during development and reduced satiety [60,61]. The relationships between the development of obesity and the genes or chromosomal regions involved in the development of these syndromes require to be fully clarified. Moreover, there are several symptoms in common among the various syndromes; therefore, further studies are fundamental to differentiate the various syndromic forms for early diagnosis of and specific treatment for each disease.

## 5. Polygenic Obesity

Polygenic obesity is due to the simultaneous presence of DNA variation in multiple genes and interaction with environmental factors; previous studies have shown that the specific set of polygenic variants relevant to obesity in one individual will hardly correspond to polygenic variants in another obese subject, because of inter-individual heterogeneity [12,62].

Currently, two gene variants, i.e., of MC4R, already known as the most common cause of monogenic obesity, and FTO-associated gene, have been identified, with small but replicable effects on body weight. Further studies are fundamental to confirm the involvement in the regulation of body weight of a third gene, i.e., insulin-induced gene 2 (INSIG2) [46,63,64,65,66].

In conclusion, further investigations are crucial to disclose the genes involved in the pathogenesis of obesity, as well as all the molecular pathways involved in genetic obesity. Moreover, new forthcoming data might permit the design of drugs effective in preventing and counteracting this complex pathology.

## 6. Epigenetic Mechanisms and Obesity Risk

Epigenetics is one of the promising areas of future medical research that can influence the way people develop and manage diseases [67]. Epigenetics is the study of heritable changes in gene expression that result in phenotype variations without changes in the DNA sequence. Factors such as age, diet, environment, and disease status can influence epigenetic changes [68,69]. Epigenetics represents a control layer to determine which genes are down-regulated and which genes are up-regulated in specific cells of the body [70,71]. The epigenetic mechanisms currently identified are DNA methylation, posttranslational modification of histone proteins, and non-coding RNA (ncRNA)-associated gene silencing [70,71]. The risk of developing obesity or diseases such as type 2 diabetes can be influenced by diet and environmental factors. Therefore, numerous scientists are interested in studying the implication of different epigenetic processes in the pathogenesis of these diseases [72,73,74]. Although it is known that external factors can cause cell-type dependent epigenetic changes, the regulation of these processes, as well as the extent of the changes, the types of cells in which they occur, and the most predisposed individuals, remain unknown. Several studies showed that a healthy diet can positively influence the individual epigenetic profile; several data, indeed, indicate that normal-weight and non-diabetic people have epigenetic profiles different from those of obese and diabetic subjects [75]. Non-nutritional risk factors associated with obesity such as hyperglycemia, inflammation, endocrine disruptors, hypoxia, and oxidative stress, as well as nutritional factors, appear to be involved in epigenetic modifications that influence adipogenesis and insulin sensitivity [73]. The emergence of epigenetic profiles that will contribute to the creation of individual microbiomes and susceptibility to the development of obesity, inflammation, and a cluster of other disorders, is already observed in fetal age and is influenced by maternal diet during pregnancy. Indeed, recent evidence has shown that maternal nutrition is closely associated with obesity in the offspring, indicating that obesity has an evolutionary origin [76,77]. Currently, studies on animal models demonstrate an effect on the epigenome of early childhood nutritional exposure and long-term metabolic health of newborns; on the contrary, human data are still limited, although several studies have provided clear evidence that exposure to unhealthy nutrition during pregnancy is associated with methylation changes in newborns, resulting in modification of the adult phenotype [78,79]. In 2012, Begum et al. studied the implication of malnutrition in pregnancy on the development of obesity and type II diabetes in a sheep model. The authors found that sheep that had suffered from moderate maternal malnutrition showed a decrease in the methylation of the promoters of the proopiomelanocortin receptor (POMCR) and glucocorticoid receptor (GR) in the fetal hypothalamus, which could lead to a long-term altered regulation of energy balance in the newborn [80]. In addition, epigenetic changes induced by unhealthy feeding can directly affect adulthood. Animal model studies, indeed, have shown that a diet rich in fats and carbohydrates, with a consequent increase in body weight, can be associated with changes in DNA methylation patterns, which affect the promoter region of several genes involved in energetic homeostasis and obesity, such as LEP, NADH dehydrogenase (ubiquinone) 1 β subcomplex subunit 6 (NDUFB6), and FASN. Recent evidence has suggested that nutrients involved in the methyl group metabolism can significantly influence epigenetics [81,82]. The genetic and epigenetic machinery regulates body weight homeostasis; it appears, indeed, that the epigenetic regulation of gene expression represents an important factor in the inflammatory process, with the consequent secretion of adipokines, such as leptin, and cytokines, such as TNF, caused by the increase in adipose tissue associated with obesity.

In 2009, Campion et al. assessed whether epigenetic regulation of the human TNF promoter by cytosine methylation could be involved in the predisposition to lose weight after a balanced low-calorie diet, constituting the first step towards a personalized diet based on epigenetic criteria. Their data showed that TNF promoter methylation was a good biomarker for predicting diet-induced weight loss [83]. These results were confirmed in 2011 by Cordero et al., who suggested that the methylation levels of leptin and TNF-alpha could be used as epigenetic biomarkers with regard to the response to a low-calorie diet. Moreover, it is possible to predict the susceptibility to weight loss, as well as some comorbidities, such as hypertension or type 2 diabetes [84]. Further studies are required to identify and understand the role of epigenetic signals that could be used as early predictors of metabolic risk. At the same time, these data will permit the development of drugs or dietary treatments to improve the prevention and therapy of obesity.

## 7. Conclusions

The expression of genes, in particular, those related to the leptin–melanocortin system, fundamental for the regulation of energy homeostasis, involved in the development of obesity, is modulated by genetic factors and environmental factors. It is important to clarify the pathways involved in the pathogenesis of obesity induced by genetic mutations and to design specific therapeutic strategies. Furthermore, new scientific advancements are essential to identify molecules able to interfere with molecular pathways and control the progression of symptoms. It will be useful to manage molecules able to cross the blood–brain barrier and exert their therapeutic effects on central neurons. Currently, because genetic mutations do not fully clarify the inheritance of obesity, other forms of variation, such as that due to epigenetics, must be taken into the account. Complex interactions between food components and epigenetic mechanisms, such as histone modifications, DNA methylation, non-coding RNA expression, and chromatin remodeling factors, lead to the dynamic regulation of gene expression that controls cell phenotype. In particular, predisposition to obesity and weight loss outcomes have been repeatedly associated with changes in epigenetic patterns. Nutritional influence on epigenetic regulation can occur at all ages, although the perinatal period is the moment of maximum phenotypic plasticity.

Further studies are required to clarify the pathogenic mechanisms underlying obesity, which is fundamental for the development of effective treatments.

## Figures and Tables

**Table 1:** Obesity induced by genetic factors.

Genetic Obesity		
Non-Syndromic Forms		Syndromic Form
Monogenic Obesity	Polygenic Obesity	Chromosomal or Pleiotropic Forms
Leptin deficiency LEPR mutation POMC deficiency PC mutation MC4R mutation SIM1, BDNF, TRKB mutations	MC4R mutation FTO mutation INSIG2 mutation	Bardet Biedl syndrome Prader Willi syndrome Alstrom syndrome Smith–Magenis syndrome

The genetic factors can be classified into monogenic, i.e., caused by a single gene mutation, syndromic, i.e., associated with other phenotypes, such as abnormalities of neurological development or organs/system malformations, and polygenic, i.e., caused by the mutation of a large number of genes. LEPR (leptin receptor), POMC (proopiomelanocortin), PC (prohormone convertase), MC4R (melanocortin receptor), SIM 1 (SIM BHLH Transcription Factor 1), BDNF (brain-derived neurotrophic factor), TRKB receptor (tropomyosin-related kinase B), FTO (fat mass and obesity), and INSIG2 (insulin-induced gene 2).

## Abbreviations

ACTH	Adrenocorticotrophic Hormone
AGRP	Agouti Related Protein
ARC	Arcuate Nucleus
BDNF	Brain-derived Neurotrophic Factor
BMI	Body Mass Index
cAMP	cyclic Adenosine Monophosphate
CCK	Cholecystokinin
CNS	Central Nervous System
FTO	Fat mass and obesity
GHSR	Growth Hormone Receptor
GLP1	Glucagon-like peptide 1
INSIG2	Insulin-induced Gene 2
IRS	Insulin Receptor Substrate
LEP	Leptin
LEPR	Leptin Receptor
MC4R	Melanocortin Receptor
ncRNA	non-coding RNA
NPY	Neuropeptide Y
PC	Prohormone Convertase
PI3K	Phosphatidylinositide-3 Kinase
POMC	Proopiomelanocortin
PYY	Tyrosine- Tyrosine Polypeptide
SIM1	Sim BHLH Transcription Factor1
TRKB	Tropomyosin-Related Kinase B
WHO	World Health Organization
α-MSH	α-melanocyte stimulating hormone
